# Neuroprotective effects of metformin on cerebral ischemia‐reperfusion injury by regulating PI3K/Akt pathway

**DOI:** 10.1002/brb3.2335

**Published:** 2021-09-02

**Authors:** Cailian Ruan, Hongtao Guo, Jiaqi Gao, Yiwei Wang, Zhiyong Liu, Jinyi Yan, Xiaoji Li, Haixia Lv

**Affiliations:** ^1^ Department ofMedicine Xi'an Jiaotong University No. 76 Yanta West Road Xi'an shannxi 710061 P. R. China; ^2^ College of Medicine Yan'an University Yan'an shannxi 716000 P. R. China

**Keywords:** cerebral ischemia‐reperfusion, inflammation, metformin, PI3K/Akt

## Abstract

Metformin (Met) is a commonly used drug in the treatment of type 2 diabetes. Currently, it has been found that Met can effectively reduce the incidence of stroke and exert anti‐inflammatory effects. However, its role in ischemia‐reperfusion (I/R)‐induced nerve injury remains unclear. This study aims to investigate the neuroprotective effects of Met in I/R‐induced neuron injury as well as the underlying mechanism. A middle cerebral artery occlusion (MCAO) model was established in Sprague Dawley (SD) rats, which were then treated with different doses of Met. Neurological deficits of rats were measured at different times post‐surgery. TTC staining was done to observe the volume of cerebral infarction. HE staining was performed to observe pathological changes of brain tissues. Immunohistochemistry was performed to observe the expression of inflammatory factors in the cerebral tissues. qRT‐PCR method was used to detect the relative expression of PI3K, Akt mRNA in cells after 24 h of drug action. Western blot method was used to detect the expression of PI3K, p‐PI3K, Akt, and p‐Akt in hippocampus. What is more, in vitro experiments were performed on BV2 microglia to verify the role of Met against oxygen‐glucose deprivation (OGD). As a result, Met dose‐dependently attenuated neurological deficits and neuronal apoptosis. Besides, Met administration also significantly reduced BV2 cells apoptosis and inflammatory response. Mechanistically, Met inactivated PI3K/Akt pathway induced by I/R and OGD, while it upregulated PI3K. In conclusion, Met protected rats from cerebral I/R injury via reducing neuronal apoptosis and microglial inflammation through PI3K/Akt pathway.

## Abstract 2

This study investigated the new use of metformin (Met), a conventional treatment for type 2 diabetes mellitus, and the neuroprotective effect of Met on cerebral ischemia‐reperfusion injury of brain by inhibiting inflammation, oxidative stress, apoptosis, etc. It belongs to old medicine and new use and has important research value.

## 1 INTRODUCTION

Cerebrovascular diseases are the main cause of death among the elderly worldwide; among them, ischemic brain injury accounts for about 70% to 80% of all cerebrovascular patients, seriously threatening human health (Kim et al., 2020). At present, intravenous injection of recombinant tissue plasminogen activator (r‐TPA) and intra‐arterial therapy have been widely used clinically and have shown to reduce the risk of disability (Chen et al., 2002). However, the therapies may further aggravate neuronal death and neurological dysfunction at the same time (Wang et al., 2020). These side reactions involve a variety of pathophysiological mechanisms, among which inflammatory mechanisms have received increasing attention (Jung et al., 2020). Therefore, understanding the inflammatory mechanism after a cerebral ischemia‐reperfusion (I/R) injury is helpful to improve its treatment.

The results of epidemiological studies suggest that Met, as a first‐line drug for the treatment of type 2 diabetes, not only can play a hypoglycemic role, but can also reduce the risk of stroke in diabetics (Fujihara et al., 2021, Matsuda et al., 2019). Met plays an important role in various diseases through different mechanisms. For example, Tokuyama team research shows, after 2 h of transient middle cerebral artery ischemia, three consecutive days of intraperitoneal administration, three times a day, it can effectively reduce the volume of cerebral infarction after cerebral ischemia (Han et al., 2005). Mc Cullough team used a 60‐min transient middle cerebral artery occlusion (MCAO) model; intraperitoneal injection of Met was started 24 h after ischemia, once a day, it was found that after 21 days of injection, Met can reduce brain atrophy (Dai et al. (2016). Researchers believe that Met may inactivate PI3K inhibition of inflammatory response after ischemia (Wahl et al., 2019). Whether the mechanism of Met in I/R injury is related to PI3K, and how it is related to PI3K regulation remains to be investigated. Therefore, in the past 10 years, the research on whether Met can be used in therapeutic cerebral I/R injury has gradually become a hot spot.

The purpose of this study was to investigate the neuroprotective effects of Met on ischemic‐induced nerve injury. Rats suffering from cerebral I/R injury were treated with Met and we found that Met dose‐dependently ameliorated neurological deficits of rats and neuronal damage in the brain lesions. Moreover, Met increased the expression of PI3K and Akt. Therefore, we speculated that Met could exert neuroprotective effect via modulating the PI3K/Akt signaling pathway.

## 2 METHODS

### 2.1 Animals

All experiments were approved by the Animal Ethics Committee of School of Medicine, Yan'an University (SXYA202001). All experiments were taken to minimize the animal suffering and reduce the number of animals; 60 male SD rats, weighing 180–220 g, were purchased from Animal Center of Air Force Military Medical University (Shanxi, China). The rats were housed in a temperature‐ and humidity‐controlled animal dormitory, with light/dark cycles lasting 12 h.

### 2.2 Establishment of experimental middle cerebral artery occlusion/reperfusion model in rats

**FIGURE 1:**
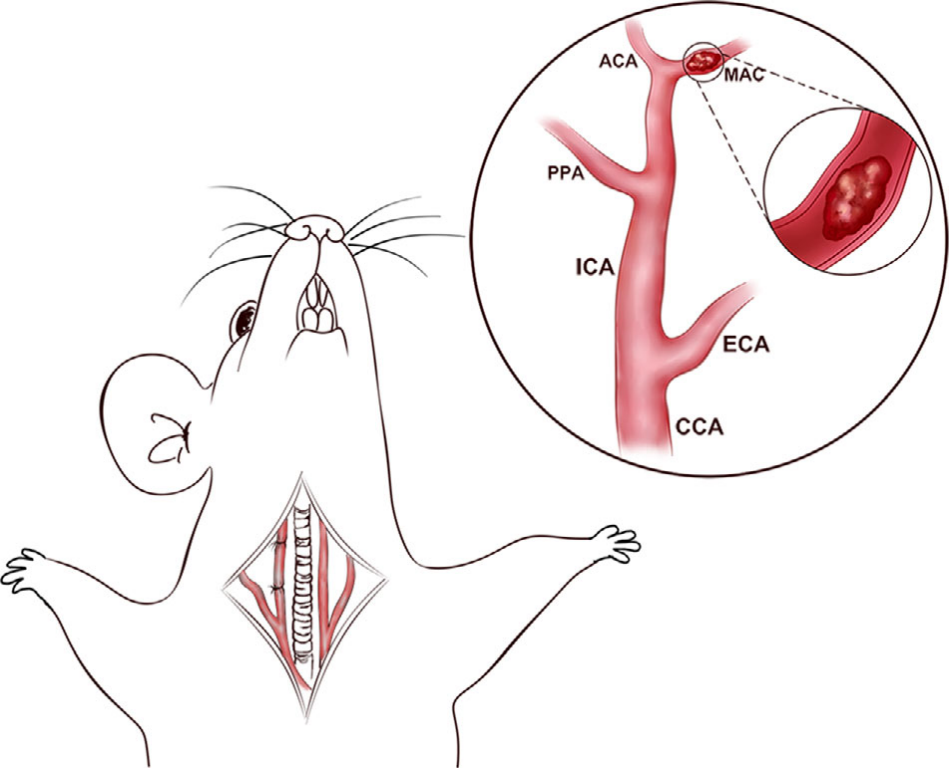
MCAO/R modeling process

A rat model of I/R injury was established using middle cerebral artery occlusion/reperfusion (MCAO/R) (Orihuela et al., 2016). The rats were anesthetized with 4% sodium pentobarbital (40 mg/kg) by intraperitoneal injection. Sterilization of the neck was done with an ethanol cotton ball and a 1‐cm incision was opened along the neck with surgical scissors. Blunt dissection was operated at the submandibular gland under a stereoscopic microscope, and the right common carotid artery, external carotid artery, and internal carotid artery were dissociated. The common carotid artery was fastened with a silk thread. We used a silk thread to tie a loose knot at the bifurcation of the external carotid artery and common carotid artery, ligated the distal end of the external carotid artery with a silk thread, and fused the external carotid artery with an electrocoagulation pen. We tightened the internal carotid artery with a silk thread, cut a small hole into the external carotid artery with micro‐ophthalmic surgical scissors, inserted the threaded plug down through the small hole into the external carotid artery, and loosened the knot on the internal carotid artery. Then we inserted the suture slowly into the internal carotid artery until resistance was felt (Figure 1). Before the operation, the 60 rats were randomly divided into five groups (each group had 15 rats): Sham group, MCAO/R group, MCAO/R + Met 3 mg/kg/d group, MCAO/R + Met 10 mg/kg/d group, and MCAO/R + Met 30 mg/kg/d group. During reperfusion, 0.9% NaCl saline (sham and MCAO/R group), Met (3, 10, 30 mg/kg/d) were intraperitoneally, respectively, it lasted for 7 days.

### 2.3 TTC staining

For the quantification of infarct volumes, the brains were removed after anesthesia. After being removed and frozen at −20°C for 5 min, the brains were sliced into five slices (2 mm thick coronal sections) and were stained with a 2% solution of TTC (Sigma Co., Ltd., St. Louis, MO) at 37°C for 10 min in the dark. The slices were then fixed with 4% neutral paraformaldehyde for 24 h before being photographed. Infarct areas were calculated by using Image J image analysis software (http://rsb.info.nih.gov/ij/), and were multiplied by slice thickness to give the infarct volume.

### 2.4 Water maze experiment

The Morris water maze experiment was used to observe the behavioral changes in animals. Specific steps refer to the method of Yao et al. (Kumar et al., 2017). A circular stainless‐steel pool with a diameter of 100 cm and a height of 50 cm was divided into four quadrants. A circular hidden platform with a diameter of 9 cm and a height of 27 cm was placed in the center of the target quadrant. The platform was hidden 1 cm below the water. A camera was used to record the movement trajectory of rats above the pool, and the Ethovision XT monitoring system was used for analysis. The rats in different groups were randomly placed into a quadrant (W, E, S, or N). The escape latency to the platform, the time spent in the targeting quadrant, as well as the times crossing the platform were recorded, respectively, and tested for three trials.

### 2.5 Modified neurological severity score

After 24 h of I/R injury, modified neurological severity scores (mNSS) were used to evaluate the neurological functions of the rats. mNSS includes motor (muscle status, abnormal movement), sensory (visual, tactile, and proprioceptive), balance beam, and reflex tests. Professionally trained researchers who did not know the experimental groupings conducted the evaluation. The lowest total score was 0, indicating complete normality and no neurological deficits; the highest score was 18, which represents loss of consciousness or death in rats.

### 2.6 Cell culture and the establishment of OGD cell model

Microglial cell line BV2 was purchased from the Cell Center of Chinese Academy of Sciences (Shanghai, China). Cells were cultured in DMEM (Thermo Fisher Scientific, MA, USA) with 10% fetal bovine serum (FBS) (Thermo Fisher Scientific, MA, USA) and 1% penicillin/streptomycin (Invitrogen, CA, USA) at 37°C and with 5% CO_2_, 95% air. Cells were trypsinized with 0.25% trypsin (Thermo Fisher HyClone, Utah, USA) and sub‐cultured during the logarithmic growth phase. To establish an OGD cell model, BV2 cells at logarithmic growth phase were cultured in D‐Hank's medium and then incubated under hypoxic conditions (temperature 37°C, and atmosphere 95% N_2_ and 5% CO_2_). After 12 h of incubation, BV2 cells were then cultured in normal culture medium at 37°C and with 5% CO_2_ and 95% air.

### 2.7 Flow cytometry

BV2 cells were seeded on 6‐well plates for 24 h. After being treated with OGD and Met, the cells were harvested and suspended in PBS, and then treated for 30 min with FITC‐labeled Annexin V and PI (Nanjing KeyGen Biotech. Co. Ltd.) for 10 min at room temperature in the dark. Next, the cells were incubated with binding buffer (400 μl). Finally, the flow cytometry (EMD Millipore, Billerica, MA, USA) was used to test cell apoptosis.

### 2.8 Drug treatment

Met (Sigma‐Aldrich, Shanghai, China) and EX 527 (Abcam, ab141506, Shanghai, China) were dissolved in 1% dimethyl sulfoxide (DMSO, Sigma‐Aldrich) with 0.1 M phosphate buffer solution (PBS). Rats were intraperitoneally given 3, 10, 0 mg/kg/d Met or DMSO (0.1 M PBS + 1% DMSO). The total volume was 100 μl. In the in vitro experiment, BV2 cells were treated with Met (3, 10, 30 μg/ml) for 24 h after undergoing OGD/R for 24 h.

### 2.9 Immunohistochemistry

Immunohistochemistry was used to detect neuronal apoptosis and microglia activation. The fixed tissue was dehydrated, embedded, and sliced according to conventional methods, and baked at 60°C for 1 h. The sections were dewaxed in xylene I, xylene II, and xylene III for 5 min, respectively. After that, the sections were placed successively in anhydrous ethanol, 95% ethanol, and 75% ethanol for 5 min, rinsed with tap water for 5 min, sliced and placed in a citrate buffer for microwave repair for 10 min, cooled at room temperature, added with 0.3% hydrogen peroxide, and incubated in a wet box for 15 min in the dark. Next, the sections were incubated with the antibody anti‐Caspase‐3 (1:300, ab2302, Abcam) or anti‐Iba1 (1:200, ab5076, Abcam) at 4°C overnight. The working solution of HRP‐labeled secondary antibody was added at 37°C. Then, the sections were incubated with a peroxidase‐conjugated polymer for 30 min, and a DAB (Beyotime Institute of Biotechnology, Shanghai, China) system was used for detection. Finally, we observed and analyzed the average gray level of positive expression sites using Image‐Pro Plus software (Media Cybernetics, USA).

### 2.10 Caspase‐3 activity detection

Caspase‐3 activity in the brain lesions was measured using Caspase3 activity assay kit (Cell Signaling Technology, Inc., Shanghai, China). Briefly, the tissues were homogenized in an appropriate lysis buffer at 4°C. Next, the lysate was centrifuged and the protein content of the supernatant was quantified. The Caspase3 viability was determined by a microplate reader (Bio‐Rad) at 450 nm.

### 2.11 qRT‐PCR

The total RNA of cells was routinely extracted using TRIzol reagent (Ambion, Life Technologies, USA). A cDNA reverse transcription kit was used to reverse transcription reaction according to the operation procedures. Next, the reversed cDNA was amplified using a quantitative PCR reaction system, which contained 500 ng cDNA, 250 nmol/L upstream and downstream primers, and 12.5 μl of 2 × SYBR Green Realtime PCR Master Mix (TOYOBO, Osaka, Japan). The primers were as follows: (Table 1) Statistics were performed using the 2 ^−ΔΔCt^ method. Each experiment was repeated three times.

### 2.12 Western blot

Total protein of the cells and tissues was extracted using RIPA lysate. Concentration of the protein was measured by the BCA method. Then the total protein was separated by SDS‐PAGE electrophoresis at a constant voltage of 100 V for 60 min. Next, the separated protein was electroporated into the PVDF membranes. After being blocked by 5% skimmed milk at room temperature for 2 h, the membranes were incubated with primary antibodies PI3K (Abcam, ab110304, 1:1000), Akt (Abcam, ab16502, 1:1000), p‐Akt (Abcam, ab86299, 1:1000), Caspase3 (Abcam, ab13847, 1:1000), Bcl2 (Abcam, ab32124, 1:1000), at 4°C overnight. The next morning, the membranes were washed by TBST and then incubated with secondary antibodies for 1 h at room temperature. Finally, the immunoblots were exposed by Chemistar™ High‐sig ECL Western Blotting Substrate (Tanon Science & Technology Co., Ltd.). β‐actin was used as the internal reference of the detected protein. Each experimental group was repeated three times.

### 2.13 CCK‐8 assay

BV2 cells were seeded into 96‐well plates with 100  μl per well of complete medium. Cells were injured by 20 μM Met, and treated with different concentrations of BAY 73–6691 (5, 10, 20 μg/ml). CCK‐8 (NeoBioscience, Shanghai, China) was utilized to evaluate cell viability according to the manufacturer's instruction. The absorbance was recorded at 450 nm using a microplate reader (BioTek, USA).

### 2.14 Data statistics

SPSS23.0 statistical software (SPSS Inc., Chicago, IL, USA) was used for analysis. The measurement data were expressed as mean ± standard deviation ($\bar{x}$ ± *s*). *t*‐test was performed to compare the mean of the two sample groups. One‐way analysis of variance was used to compare the mean of multiple samples. *p* < .05 was considered statistically significant.

## 3 RESULTS

### 3.1 Met reduced infarct volumes in MCAO/R injury rats

**FIGURE 2:**
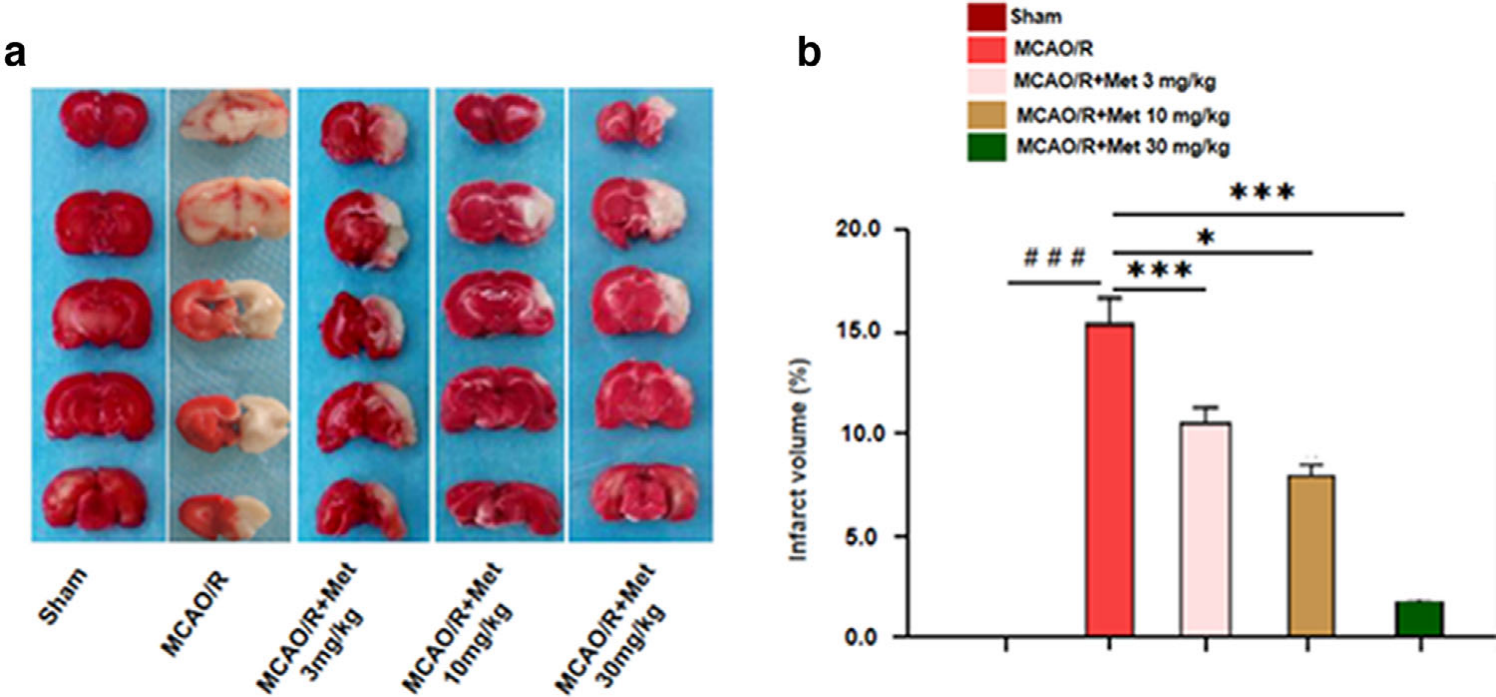
Met reduced infarct volumes in MCAO/R injury rats. (a, b) The infarct volume was detected by TTC staining. (a, b) TTC staining detection of cerebral infarction volume in rats versus Sham group: ^＃^ *p* < .05, ^＃＃^ *p* < .01, ^＃＃＃^ *p* < .001; versus MCAO/R group: *p < .05, **p < .01, ***p < .001

Infarct volumes were obtained when ischemia was given for 2 h and reperfusion for 24 h. The white area represented infarcted tissues and red represented normal tissues. TTC staining results show that the volume of cerebral infarctions significantly increased, compared with the Sham group (*p* < .001); Met (3 mg/kg/d, 10 mg/kg/d, 30 mg/kg/d) reduced the infarct volume compared with the MCAO/R group (*p* < .001, *p* < .001) (Figure 2a,b). Moreover, the cerebral infarction volume of the Met 30 mg/kg/d group was significantly reduced, suggesting that the reduction of the cerebral infarction volume by Met is positively correlated with the measurement.

### 3.2 Neuroprotective effect of Met on MCAO/R injury rats

**FIGURE 3:**
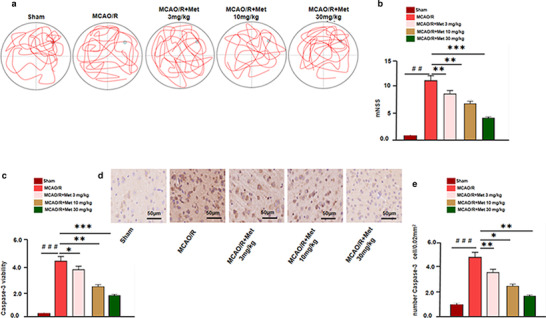
Neuroprotective effect of Met on MCAO/R injury rats. (a) Morris water maze experiment was assessed 14 days after brain injury and the representative images of Morris water maze in different groups are as shown. (b) mNSS was used to evaluate the neurological damage of rats in each group. (c) Caspase3 activity in the brain lesions were detected using a Caspase3 kit. (d, e) Immunohistochemistry was conducted to detect apoptotic cells marked by Caspase3 and the number of Caspase3 positive cells was counted versus the Sham group: ^＃^ *p* < .05, ^＃＃^ *p* < .01, ^＃＃＃^ *p* < .001; versus MCAO/R group: *p < .05, **p < .01, ***p < .001

In order to further verify the effect of Met on cerebral I/R injury, we established a model of cerebral I/R injury in rats, and treated the rats with Met. The neurological function defects of rats were assessed through water maze experiments, including spatial learning and memory, as well as motor function. The results showed that Met dose‐dependently promoted the spatial learning and memory recovery of I/R rats (Figure 3a). mNSS score displays that Met attenuated neurological deficits of the I/R rats (Figure 3b). Immunohistochemistry results show that Met significantly decreased the expression of Caspase‐3 apoptosis‐related proteins (Figure 3c–e). These results show that Met can reduce mNSS score by inhibiting the apoptosis of nerve cells, and realize the neuropenetration effect on I/R‐damaged rats.

### 3.3 Met inhibited I/R‐mediated inflammatory response

**FIGURE 4:**
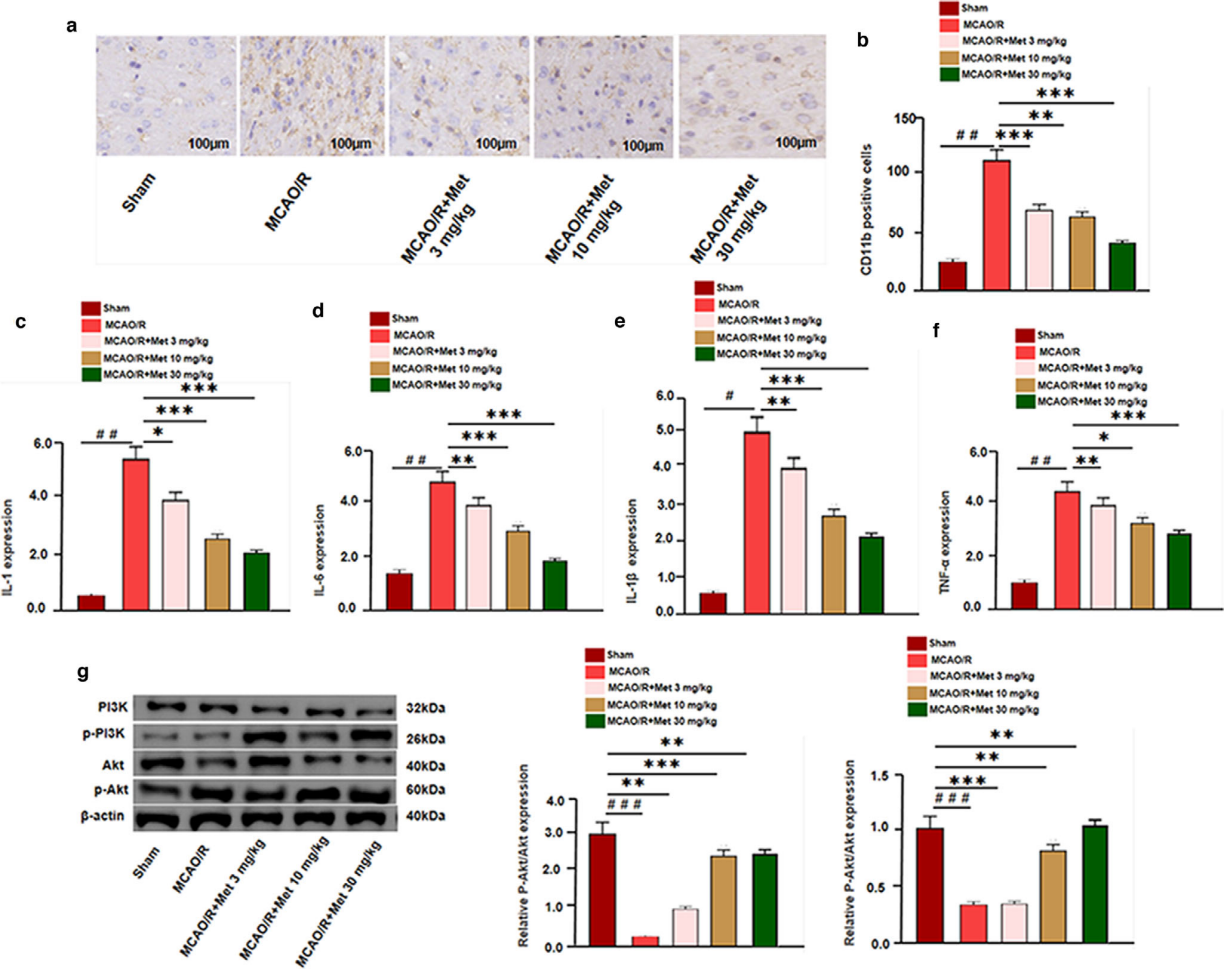
Neuroprotective effect of Met on MCAO/R injury rats. (a, b) The infarct volume was detected by TTC staining. (a–f) Immunohistochemistry was used to examine microglia activation (marked by CD11b). (g) Western blot and qRT‐PCR were used to analyze the relative expression of IL‐1, IL‐6, IL‐1β, TNF‐α, p‐PI3K, and p‐Akt in each group versus the Sham group: ^＃^ *p* < .05, ^＃＃^ *p* < .01, ^＃＃＃^ *p* < .001; versus MCAO/R group: *p < .05, **p < .01, ***p < .001

Met has certain neuroprotective effects on MCAO/R rats. To study the neuroprotective mechanism, we detected BV2 cells activation (labeled by CD11b) and the expression of inflammatory factors including IL‐1, IL‐6, IL‐1β, and TNF‐α. Immunohistochemistry results show that the number of CD11b BV2 cells, as well as IL‐1, IL‐6, IL‐1β, and TNF‐α expression, were all dramatically upregulated in the brain lesions after I/R injury (Figure 4a–f). Under the treatment of Met, CD11b‐positive cells were remarkedly diminished and the expression of IL‐1, IL‐6, IL‐1β, and TNF‐α were also decreased (Figure 4a–d). In order to further explore the anti‐inflammatory mechanism of Met, we also studied the effect of Met on PI3K and Akt expression. Western blot results showed that Met attenuated the phosphorylated level of PI3K and Akt (p‐PI3K, p‐Akt) which was activated by I/R. Moreover, Met dose‐dependently promoted the protein level of PI3K (Figure 4g). Therefore, we speculated that Met can inhibit microglia‐induced inflammation following I/R injury, and probably through PI3K‐mediated Akt pathway.

### 3.4 Met inhibited the autophagy of BV2 cells induced by OGD/R

**FIGURE 5:**
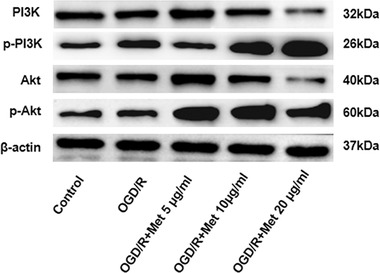
Western blot detection of relative expression of autophagy‐related proteins

When the cerebral blood oxygen supply is insufficient, the region sensitive to ischemia and hypoxia will activate the autophagy pathway, and moderate autophagy can play a protective role in ischemia and hypoxia. However, the excessive occurrence of autophagy will cause neuronal damage. The results show that, compared with the control group, the relative expression of LC3 and Beclin‐1 protein in the brain injury tissue of the OGD/R group was significantly increased, and the relative expression of p62 protein was significantly reduced (*p* < .05). Compared with OGD/R group, the relative expression of LC3 and Beclin‐1 protein in Met (5, 10, 20 μg/ml) group was significantly decreased, and the relative expression of p62 protein was significantly increased (*p* < .05), and positive correlation with dose was observed (Figure 5). (Table 2).

### 3.5 Met inhibited the apoptosis of BV2 cells induced by OGD/R

**FIGURE 6:**
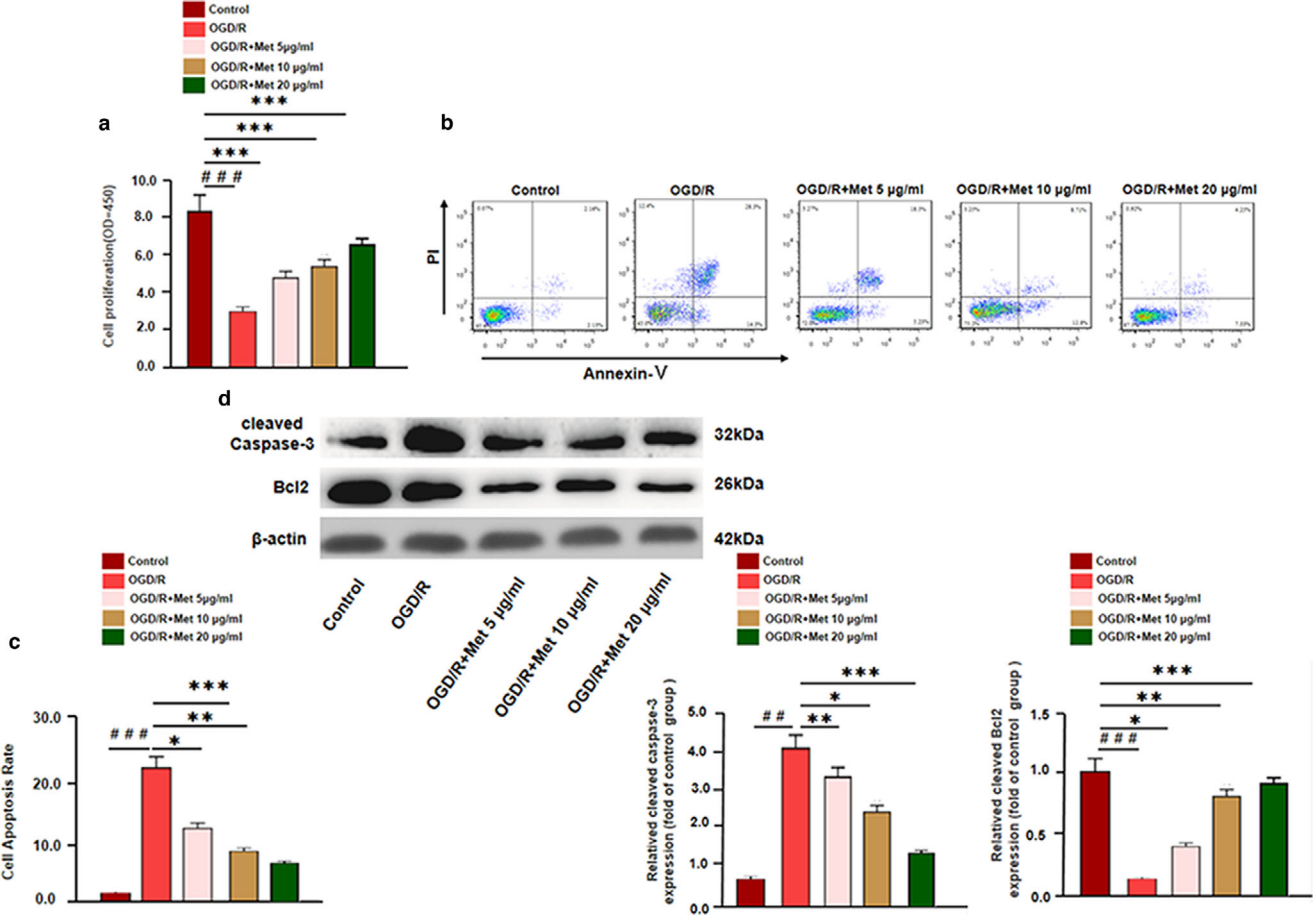
Effect of cell experiment Met anti‐apoptotic effect. BV2 cells were pre‐treated by OGD/R for 24 h, and then treated with different doses of Met (5, 10, 20 μg/ml) for 24 h. (a) The viability of BV2 cells were detected by CCK8 assay. (b, c) Flow cytometry was used to examine the apoptosis of BV2 cells. (d) Western blot was used to detect apoptotic proteins, including Caspase‐3 and Bcl2 versus control group: ^＃^ *p* < .05, ^＃＃^ *p* < .01, ^＃＃＃^ *p* < .001; versus OGD/R group: *p < .05, **p < .01, ***p < .001

In order to confirm the neuroprotective effects of Met, an in vitro model of I/R injury on BV2 cells was constructed by OGD/R. The viability of BV2 cells was detected by CCK8 assay, which showed that OGD/R induced obvious viability decrease, while Met treatment enhanced BV2 cells viability in a dose‐dependent manner (compared with OGD/R group) (Figure 6a). Moreover, the apoptosis of BV2 cells were also determined. The results of flow cytometry and western blot showed that compared with the con group, the apoptotic rate and expression of Caspase‐3 were markedly increased, and Bcl2 was downregulated under OGD/R treatment. However, Met significantly relieved the apoptosis of BV2 cells induced by OGD/R (Figure 6b–d). Therefore, the above results indicated that Met exerted neuroprotective effects against OGD/R.

### 3.6 Met downregulated OGD/R‐mediated microglial inflammation

**FIGURE 7:**
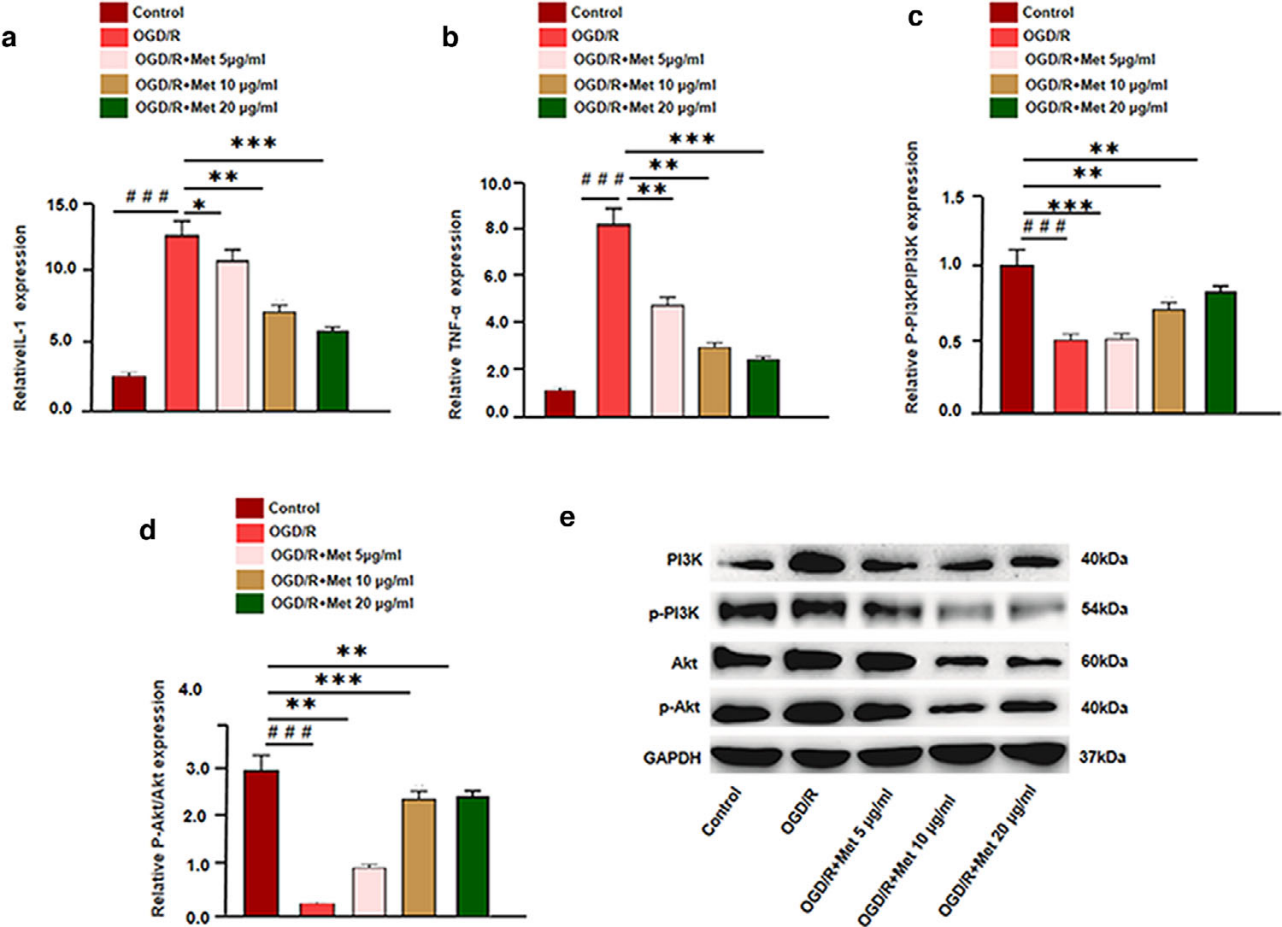
Met downregulated OGD/R‐mediated microglial inflammation. (a, b) qRT‐PCR detects the expression of inflammatory factorsIL‐1, TNF‐α after Met intervention in different doses. (c–e) Western blot and qRT‐PCR was used to analyze the relative expression of PI3K, Ak, p‐PI3K, and p‐Akt in each group versus control group: ^＃^ *p* < .05, ^＃＃^ *p* < .01, ^＃＃＃^ *p* < .001; versus OGD/R group: ^*^ *p* < .05, ^**^ *p* < .01, ^***^ *p* < .001

We treated BV2 microglia under OGD/R environment with Met, thus exploring the anti‐inflammatory effects of Met. The results showed that OGD/R markedly promoted the expression of inflammatory cytokines, including IL‐1β and TNF‐α. (Figure 7a,b). Next, the expression of PI3K and Akt was detected. We found that compared with the control group, the level of PI3K mRNA and protein were significantly reduced after OGD/R treatment, while phosphorylated level of PI3K and Akt was increased (Figure 7c,d). However, Met reversed OGD/R‐induced injure by promoting PI3K expression and inactivating Akt pathway (Figure 7e). Consequently, the data verified that Met had anti‐inflammatory effects by upregulating PI3K. What is more, this experiment suggests that the effect of Met on the inflammatory response of BV2 cells induced by OGD/R was positively correlated with the dosage of Met.

### 3.7 The anti‐inflammatory effect of Met was further verified by inhibiting the expression of PI3K

**FIGURE 8:**
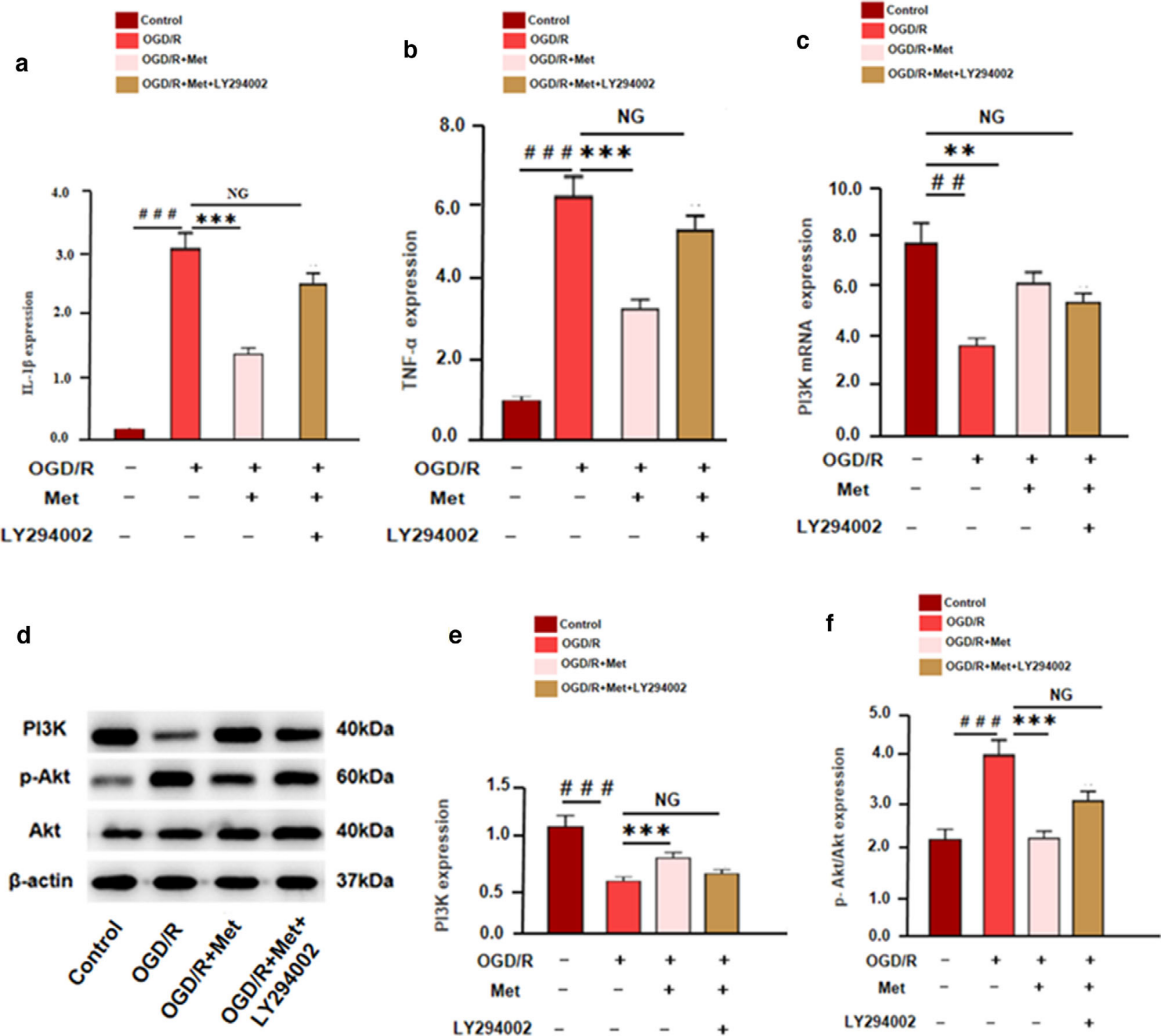
The anti‐inflammatory effect of Met was further verified by inhibiting the expression of PI3K. BV2 microglia was treated by OGD/R, Met (5 μg/ml) and/or LY294002 m (1 μM). (a, b) The expression of inflammatory cytokines, including IL‐1β and TNF‐α was tested by qRT‐PCR. (c) The expression of PI3K mRNA was detected by qRT‐PCR. (d) Western blot was used to detect the expression of PI3K, p‐PI3K, Akt, and p‐Akt versus control group: ^＃^ *p* < .05, ^＃＃^ *p* < .01, ^＃＃＃^ *p* < .001; versus OGD/R group: *p < .05, **p < .01, ***p < .001

To further confirm the role of PI3K in Met‐mediated effects, we treated OGD/R‐activated microglia with PI3K inhibitor LY294002. Then, the inflammatory response of microglia was detected. qRT‐PCR results showed that compared with OGD/R + Met group, the inflammatory cytokines (IL‐1β and TNF‐α) and phosphorylated Akt were all enhanced in the OGD/R + Met + LY294002 group, while PI3K was downregulated. However, there were no significant differences between OGD/R group and OGD/R + Met + LY294002 group in terms of the inflammatory cytokines and proteins (Figure 8a–f). Hence, the above results proved that Met exerted its effects dependently through PI3K.

## 4 DISCUSSION

Stroke, the second‐most prominent cause of death worldwide, encompasses hemorrhagic stroke, but the majority of cases are caused by arterial occlusion causing ischemic injury (Kumar et al., 2017). Stroke results in acute neuronal cell death and focal brain inflammation, which aggravates secondary brain injury by exacerbating blood–brain barrier damage, microvascular failure, brain edema, and oxidative stress (Humphris et al., 2014). Thanks to technological innovations, the clinical management of ischemic stroke has greatly advanced, notably through the use of intravenous thrombolysis and endovascular thrombectomy, which reduces disability (Drummond & Wisniewski, 2017). However, identification of pathways and molecules that participate in cerebral ischemia could reveal novel approaches to improve the clinical outcome (Xu et al., 2020). In this regard, animal models mimicking human stroke, such as the MCAO model, enable the study on the pathogenesis of cerebral ischemia (Zhao et al., 2020).

**TABLE 1:** Various related gene sequences

Name	Sequences
GAPDH	FOR:TGCTGATATGTCGTGGAG
	REV:GTCTTCTGAGTGGCAGTGAT
PI3K	FOR:AGCTGAGCGTGTGTGACAGTAT
	REV:CCGAACATACGATTGGGTAGTT
IL‐1β	FOR:GCACATCGCTCAGCAAATCG
	REV:ACAACTCCCAGGCTCCAGAC
TNF‐α	FOR:CCCAGGGAGGAGCAATACAG
	REV:GGGAGGACGCCATAACAACT

**TABLE 2:** Relative expression of autophagy‐related proteins in rats (¯x ± s, μg/ml)

Group	Beclin‐1	LC3	p62
Control	0.71 ± 0.12	0.23 ± 0.04	3.84 ± 0.14
OGD	1.72 ± 0.21^＃＃＃^	1.96 ± 0.08^＃＃＃^	0.96 ± 0.04^***^
OGD + Met 5 μg/ml	1.55 ± 0.23^**^	1.91 ± 0.16^*^	2.58 ± 0.10^*^
OGD + Met 10 μg/ml	1.16 ± 0.20^**^	1.34 ± 0.15^**^	2.96 ± 0.16^*^
OGD + Met 20 μg/ml	0.99 ± 0.09^**^	1.11 ± 0.13^***^	3.66 ±0.14^＃＃＃^

Metformin hydrochloride is a biguanidine diabetes drug, chemical name: 1,1‐ = methyl biguanidine hydrochloride, English name: metformin/ Met, Structure: 
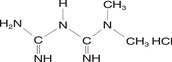
.  Cerebral I/R injury has been found to produce cytotoxicity as well as dysregulated neuroinflammation (Hou et al., 2016). In this study, we demonstrated that Met reduces neurological deficits and inflammatory responses in a MCAO/R rat model, and the underlying mechanism of Met maybe through regulating the PI3K/Akt signaling pathway. On the subject of Met as a classic hypoglycemic agent, current studies have found that it can reduce nerve damage caused by cerebral ischemia and hypoxia. For example, Met inhibits H_2_O_2_‐induced Bcl‐2 downregulation and Bax upregulation in SH‐SY5Y cells in a dose‐dependent manner, thus increasing the Bcl‐2/Bax ratio and exhibiting neuroprotective effects (Yu et al., 2016). In Alzheimer's disease (AD), Met inhibits Aβ aggregation and protects the neurons in AD (Min et al., 2020). Besides, research also showed that Met plays an important anti‐inflammatory role. For instance, it was found that in RAW 264.7 macrophages stimulated by lipopolysaccharide (LPS), Met exerts anti‐inflammatory effects partly through the PI3K/Akt signaling pathway (Min et al., 2020). In acute lung injury (ALI), Met also reduces the number of inflammatory cells and expression of pro‐inflammatory cytokines such as TNF‐α, IL‐1β, and IL‐6 (Wen et al., 2018). Presently, it was found that Met not only dose‐dependently promoted neurological rehabilitation of I/R rats, but also inhibited neuron apoptosis, microglia activation, and the expression of TNF‐α, IL‐1β, and p‐NF‐κB. These results indicated that Met has neuroprotective and anti‐inflammatory effects in cerebral I/R injury.

A series of cascade reactions, such as oxidative stress caused by cerebral ischemia and hypoxia, can lead to overactivation of autophagy and increase neuronal necrosis (Bader et al., 2020). PI3K/Akt pathway plays an important role in the regulation of autophagy. PI3K is an important signal transduction factor in cells, mainly composed of catalytic subunit P110 and regulatory subunit P85. PI3K/Akt signaling pathway plays an important biological function in the process of neuronal cell survival and apoptosis during ischemia‐reperfusion brain injury. Met activates the PI3K/Akt signaling pathway, inhibits inflammation, oxidative stress, and apoptosis, increases the actity of BV2 cells, and promotes PI3K phosphorylation to improve neurological deficit symptoms of cerebral ischemia and reperfuion.

Akt/PKB is a serine/threonine protein kinase, but also one of the important downstream target kinases in the process of PI3K signal transduction. PI3K is activated to produce PIP3, locating Akt to cell membranes, it binds to the pH region of Akt to activate Akt; this leads to a cascade of signal transduction pathways (Sarkar et al., 2020; Zeng et al., 2020). Akt activated by PI3K can activate Akt phosphorylation, regulating autophagy‐related protein Beclin1, LC3, and P62 expression, participating in the regulation of autophagy. In this experiment, the results of TTC staining showed that, after 3, 10, and 30 mg/kg Met intervention in MCAO/R injury rats, the infarct volume and mNSS score decreased, the expression of IL‐1, IL‐6, IL‐1β, TNF‐α, Beclin1, and LC3 were significantly decreased, the expression of p62, p‐PI3K, and p‐Akt were increased significantly, it was positively correlated with the dose. These results suggest that Met can inhibit apoptosis and inflammation by regulating PI3K/Akt pathway, thereby alleviating MCAO/R injury.

In conclusion, our study revealed that Met alleviates brain injury caused by I/R by modulating inflammation, autophagy, and apoptosis. The potential mechanism may be the regulation of the PI3K/Akt pathway. Collectively, the above data indicates that Met has a potential role in the treatment of brain I/R injury.

## CONFLICT OF INTEREST

The authors declare no conflict of interest.

## AUTHOR CONTRIBUTIONS

*Conceived and designed the experiments*: Cailian Ruan. *Performed the experiments*: Hongtao Guo. *Statistical analysis*: Jiaqi Gao, Yiwei Wang, Zhiyong Liu, Jinyi Yan, and Xiaoji Li. *Wrote the paper*: Cailian Ruan and Haixia Lv. All authors read and approved the final manuscript.

### PEER REVIEW

The peer review history for this article is available at https://publons.com/publon/10.1002/brb3.2335

## Data Availability

The data sets used and analyzed during the current study are available from the corresponding author on reasonable request.
